# Prospects for circumventing aminoglycoside kinase mediated antibiotic resistance

**DOI:** 10.3389/fcimb.2013.00022

**Published:** 2013-06-25

**Authors:** Kun Shi, Shane J. Caldwell, Desiree H. Fong, Albert M. Berghuis

**Affiliations:** ^1^Groupe de Recherche Axé sur la Structure des Protéines, Department of Biochemistry, McGill UniversityMontreal, QC, Canada; ^2^Department of Microbiology and Immunology, McGill UniversityMontreal, QC, Canada

**Keywords:** aminoglycosides, antibiotic resistance, drug design, kinases, structure-based, inhibitor

## Abstract

Aminoglycosides are a class of antibiotics with a broad spectrum of antimicrobial activity. Unfortunately, resistance in clinical isolates is pervasive, rendering many aminoglycosides ineffective. The most widely disseminated means of resistance to this class of antibiotics is inactivation of the drug by aminoglycoside-modifying enzymes (AMEs). There are two principal strategies to overcoming the effects of AMEs. The first approach involves the design of novel aminoglycosides that can evade modification. Although this strategy has yielded a number of superior aminoglycoside variants, their efficacy cannot be sustained in the long term. The second approach entails the development of molecules that interfere with the mechanism of AMEs such that the activity of aminoglycosides is preserved. Although such a molecule has yet to enter clinical development, the search for AME inhibitors has been greatly facilitated by the wealth of structural information amassed in recent years. In particular, aminoglycoside phosphotransferases or kinases (APHs) have been studied extensively and crystal structures of a number of APHs with diverse regiospecificity and substrate specificity have been elucidated. In this review, we present a comprehensive overview of the available APH structures and recent progress in APH inhibitor development, with a focus on the structure-guided strategies.

## Introduction

Antibiotics are fundamental to the development and practice of modern medicine. They are among the most frequently prescribed medication. They are used to treat infections but also function as prophylactics to guard against them. Moreover, they are massively used in the agriculture, serving as prophylactic agents and growth promoters in animal husbandry and fish farming (Lipsitch et al., 2002). The introduction of penicillin and streptomycin into clinical use in the 1940s marked the beginning of the golden age of antibiotic discovery. The vast majority of antibiotics in our repertoire were discovered in the following three decades by systematic empiric screening of fermentation products or chemicals for growth inhibitors of bacteria (Silver, 2011). The surge in the popularity of antibiotics led to the emergence of resistant pathogens. However, the severity of the situation was not recognized, as alternatives seemed to be perpetually available. As the discovery of new classes of antibiotics dwindled, the plight of antibiotic resistance regained its prominence. The emergence of bacterial resistance to antibiotic is inevitable but the overuse and inappropriate use of antibiotics accelerate the rate. Furthermore, high population densities in urban centers and ease of global travel facilitate the spread of resistant pathogens (Bruinsma et al., 2003; Carlet et al., 2012). As the current demographic trends toward longer lifespans and the concomitant rise in the rate of chronic and age-related illnesses (World Economic Forum, 2013), the post-antibiotic era is imminent. There is a desperate need for effective antibiotics.

The problem of antibiotic resistance has been on the forefront of public health authorities such as the World Health Organization. Its first comprehensive report on the topic was released in 2001 (World Health Organization, 2001) and antibiotic resistance was the focus for the World Health Day in 2011. Recently, the World Economic Forum listed antibiotic resistance as one of its cases in the Global Risks Report of 2013 (World Economic Forum, 2013). Furthermore, the Ontario Medical Association released a report in March 2013 addressing the issue (Ontario Medical Association, 2013). The plight of antibiotic resistance is irrefutable and the toll is alarming. Currently, about 100,000 people die from hospital-acquired infections a year in the United States (Spellberg et al., 2011). It has been estimated that antibiotic-resistant infections will impart on the healthcare system an annual cost of over $21 billion in the United States (Spellberg et al., 2011) and over $104 million in Canada (Wilson et al., 2010).

Resistance to carbapenem, a class of last-resort antibiotics derived from β-lactam, is rising and the mortality rate of infections caused by carbapenem-resistant enterobacteriaceae (CRE) is almost 50% (Borer et al., 2009). While the extent of resistance can be curbed by the prudent use of antibiotics, there remains the need for effective drugs with new mechanisms of action that are not susceptible to existing resistance mechanisms to combat such pathogens as methicillin-resistant *Staphylococcus aureus* (MRSA), vancomycin-resistant *Enterococcus* (VRE), and CRE. With no candidates in the antibiotic development pipeline, alternative strategies must be devised, such as enhancing the human immunological response with vaccines (Mishra et al., 2012) or the use of bacteriophages (Gilmore, 2012). However, the strategy that shows the most promise is the development of adjuvants to be used in combination with the existing antibiotics, either as a booster of antibiotic activity (Marks et al., 2012) or as an inhibitor of a resistant mechanism (Kalan and Wright, 2011). Inhibition of the mechanism of resistance is especially amenable for those antibiotics, such as aminoglycosides, that are rendered ineffective by enzymatic inactivation.

## Aminoglycosides

The first aminoglycoside, streptomycin, was isolated in 1943 from *Streptomyces griseus* by Albert Schatz and Selman A. Waksan (Schatz et al., 1944). It was a seminal discovery in the history of antibiotics since streptomycin was the first effective treatment for tuberculosis as well as the first useful antibiotic derived from a bacterial source. In the ensuing three decades, more aminoglycosides from actinomycetes have been identified and a number of semisynthetic variants have also been developed.

**Figure 1:**
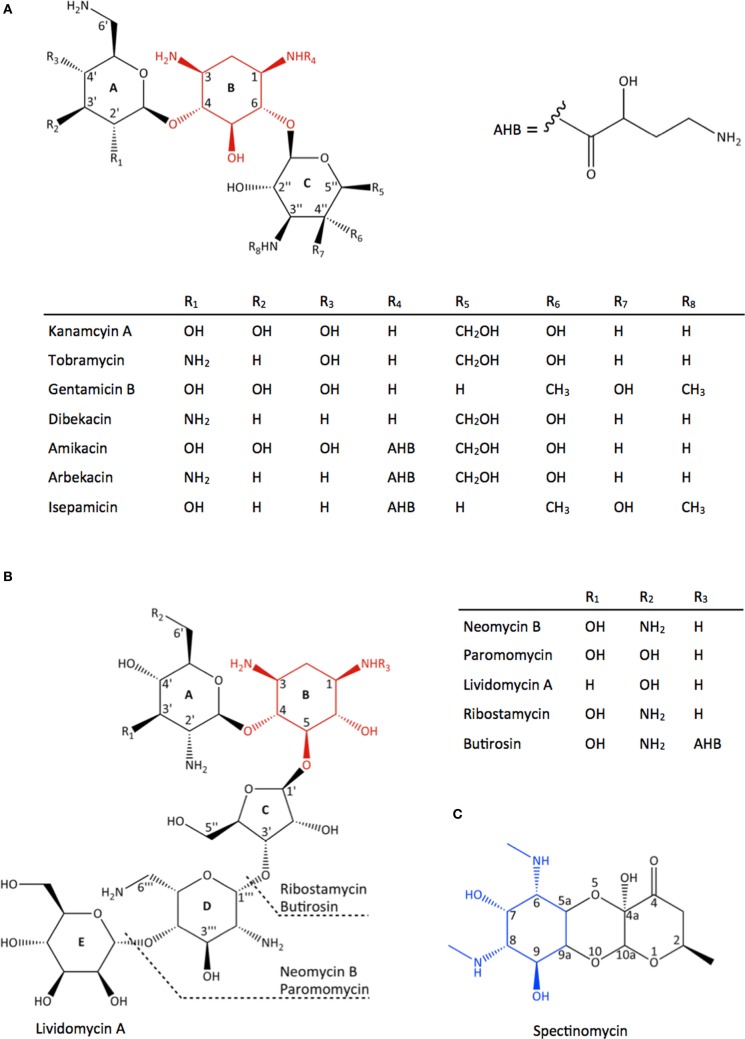
**Chemical structure of different classes of aminoglycoside antibiotics. (A)** 4,6-disubstituted aminoglycosides, **(B)** 4,5-disubstituted aminoglycosides, **(C)** spectinomycin, an atypical aminoglycoside. The 2-deoxystreptamine nucleus in **(A)** and **(B)** are highlighted in red and the streptamine nucleus in **(C)** is highlighted in blue.

Aminoglycosides encompass a large group of aminocyclitol-containing molecules that are structurally diverse, hydrophilic, and polycationic. They can be categorized into three major groups based on their structures (Figure 1). The first group, which includes streptomycin, contains a streptamine nucleus. The second group, which includes spectinomycin and hygromycin B, contains either a streptamine or a 2-deoxystreptamine nucleus and they have distinctive structures due to their fused ring systems. The third and largest group, which includes paromomycin and gentamicin, contains a 2-deoxystreptamine nucleus with amino sugar rings substituted at either positions 4 and 5 or positions 4 and 6. The 6-amino hexose ring linked to position 4 of the 2-deoxystreptamine is designated as the prime (′) or A ring and the pentose or hexose ring linked to position 5 or 6 is labeled the double prime (″) or C ring; the central 2-deoxystreptamine ring is sometimes referred to as the B ring.

Aminoglycosides target the 30S ribosomal subunit of the bacteria and interfere with protein synthesis. The three classes of aminoglycosides have different binding targets in the ribosome as well as mechanisms of action. Streptomycin binds to the 16S rRNA near a ribosomal accuracy switch, stabilizing the A-site in the *ram* or an “error-prone” state (Carter et al., 2000). The higher affinity for aminoacyl-tRNA in the *ram* state makes the binding of non-cognate tRNA more favorable and impairs the proof-reading mechanism (Karimi and Ehrenberg, 1994). Spectinomycin is unique among the aminoglycosides in that it is a bacteriostatic agent. It has been shown to inhibit the translocation of the peptidyl-tRNA from the A- to P-site (Bilgin et al., 1990). Based on the location of its binding site in the 30S ribosomal subunit, it is postulated that spectinomycin acts by sterically hindering the conformational changes or movements necessary for translocation (Carter et al., 2000). 2-deoxystreptamine aminoglycosides induce errors in protein translation by binding to the A-site of the 16S rRNA (Moazed and Noller, 1987) and trigger conformational changes that improves the stability of the binding of near-cognate aminoacyl-tRNA to the decoding center (Carter et al., 2000; Pape et al., 2000). As a result, the ribosome incorporates erroneous amino acid residues, synthesizing defective proteins, and precipitating cell death.

Aminoglycosides have been an important component in the antibiotic armamentarium due to their low cost, efficacy against both Gram-negative and some Gram-positive bacteria, their synergism with other antibiotics, as well as their pharmacokinetic and pharmacodynamic properties. Furthermore, some aminoglycosides have also been shown to be effective against protozoa (Berman and Fleckenstein, 1991) and *Neisseria gonorrhoeae* (Duncan et al., 1972). Aminoglycosides are usually used as the treatment of serious infections, such as septicemia, peritonitis, and skin infections associated with burns, caused by Gram-negative bacteria (Gonzalez and Spencer, 1998; Prayle and Smyth, 2010). In particular, tobramycin, gentamicin, and amikacin are effective treatments for chronic respiratory infections caused by *Pseudomonas aeruginosa* that plague patients with cystic fibrosis (CF) (Prayle and Smyth, 2010). Aminoglycosides are also able to target Gram-positive infections, such as endocarditis, when used in combination with a cell wall active antibiotic, e.g., β-lactams (Graham, 2002). Although streptomycin is no longer a part of the anti-tuberculosis treatment regime, kanamycin and amikacin are indicated for the treatment of multi-drug resistant tuberculosis (MDR-TB) (Caminero et al., 2010). Nowadays, aminoglycosides are more commonly reserved for second-line antibiotic therapy due to their resistance in pathogens and their nephrotoxic and ototoxic effects (Lopez-Novoa et al., 2011; Matt et al., 2012). Although the adverse side effects have been mitigated with the evolution of dosing schemes (Begg and Barclay, 1995), resistance to aminoglycosides continues to rise to alarming levels.

## Mechanisms of resistance to aminoglycosides

### Decreased intracellular concentration

Although the precise mechanism by which aminoglycosides enter the bacterial cell remains elusive, the working model of aminoglycoside uptake consists of three steps. The first step is thought to be the non-specific electrostatic interaction between the polycationic aminoglycosides and the negatively charged portions of the bacterial membrane. Subsequent steps are dependent on the membrane potential (Bryan et al., 1979; Taber et al., 1987). Hence anaerobes are intrinsically resistant to aminoglycosides (Bryan et al., 1979; Poole, 2005). Reduced accumulation of aminoglycoside can be a result of reduced uptake of the antibiotic due to alterations in the composition of the membrane surface or defects in the plasma membrane potential; alternatively, aminoglycosides can be purged from the bacterial cell by active efflux. Although relatively few bacterial drug efflux systems are capable of removing aminoglycosides due to the antibiotic's polycationic nature (Poole, 2005), active efflux is the most common mechanism of aminoglycoside resistance in lung isolates of *P. aeruginosa* from CF patients (Poole, 2011). Repeated use of aminoglycosides upregulates the production of the chromosomally encoded multidrug efflux system MexXY-OprM, leading to a pan-aminoglycoside resistant phenotype (Poole, 2011; Morita et al., 2012).

### Target modification

Alterations to the aminoglycoside binding site in the ribosome can be brought about by ribosome mutation or enzymatic modification by methyltransferases. The resulting alteration in the ribosome or the addition of a methyl group precludes the binding of the antibiotic to its target site. Soon after its clinical implementation as treatment of tuberculosis, resistance to streptomycin began to appear (Iseman, 1994). Aminoglycoside resistance to *Mycobacterium tuberculosis* is most often a consequence of mutations in the *rps*L gene encoding the ribosomal protein S12 or in the *rrs* gene encoding the 16S rRNA. A dominant resistance phenotype in mycobacteria derived from ribosome mutations can be attributed to the presence of a single *rrn* operon in this pathogen (Meier et al., 1994). For resistance to streptomycin, the most frequent mutations are detected in the *rps*L gene, substituting a lysine at codon 43 or 88 with an arginine and in the *rrs* gene in regions around nucleotides 530 and 915. An altered *rps*L or *rrs* gene accounts for 80% of streptomycin resistance in *M. tuberculosis* strains (Meier et al., 1994). Similarly, a A1400G point mutation leads to an amikacin/kanamycin resistance phenotype (Alangaden et al., 1998).

Ribosomal methylation is becoming a significant mechanism of aminoglycoside resistance in pathogens due to the high level of resistance they confer and rapid worldwide dissemination (Doi and Arakawa, 2007; Hidalgo et al., 2013). Methyltransfeases are inherent in aminoglycoside-producing actinomycetes but they were reported in 2003 having been found in *P. aeruginosa* and *Klebsiella pneumoniae* (Galimand et al., 2003; Yokoyama et al., 2003). These 16S-RMTases can be classified into two groups based on the site of modification. The N7-G1405 16S-RMTases constitute the larger group, having seven members. The methylation at G1405 in the decoding center confers resistance to 4,6-disubstituted 2-deoxystreptmaine aminoglycosides. To date, only one member of N1-A1408 16S-RMTase has been identified. A methylated A1408 is able to prevent the binding of all 2-deoxystreptamine aminoglycosides (Wachino and Arakawa, 2012).

### Enzymatic modification of aminoglycosides

By far, the most common mechanism of resistance to aminoglycosides is the covalent modification of the drug by aminoglycoside-modifying enzymes (AMEs). The addition of chemical groups prevents the binding of the aminoglycoside to the ribosome, thereby conferring resistance. Three classes of AMEs are known: acyl-coenzyme A-dependent acetyltransferases (AAC), nucleoside triphosphate-dependent nucleotidyltransferases (ANT), and nucleoside triphosphate-dependent phosphotransferases (APHs). Well over 100 AMEs have been identified to date (Ramirez and Tolmasky, 2010) and numerous members from each of the three classes have been subjects of extensive enzymatic and structural studies. The prevailing nomenclature system for these enzymes consists of a three-letter abbreviation identifying the enzyme activity, followed by the site of modification or regiospecificity in parentheses; next, the substrate spectrum is denoted by a Roman numeral and finally a lower case letter indicates the individual genes which confer identical resistance phenotypes (Shaw et al., 1993).

The success of aminoglycoside resistance via enzymatic modification of the antibiotic can be attributed to the ability of many of these enzymes to act on a range of aminoglycoside substrates, and the fact that most aminoglycosides can be inactivated by more than one AME. Their rapid dissemination is facilitated by their frequent occurrences within various mobile genetic elements, often alongside additional antibiotic-resistance factors. Moreover, mutation, recombination, and merging of genes have led to the formation of new enzyme variants with expanded substrate ranges including antibiotics unrelated to aminoglycosides (Robicsek et al., 2006). As a result, there are intense interests in exploring strategies to circumvent the deleterious effects of AMEs. In particular, the aminoglycoside phosphotransferase or kinase (APH) class of enzymes is the best-studied among the AMEs due to their broad substrate spectra, wide-ranging regiospecificity, and in particular, their ability to yield high levels of resistance (Vakulenko and Mobashery, 2003). The wealth of structural and mechanistic information amassed for APHs has made this family of enzymes a prototype for the development of novel anti-resistance compounds.

## Aminoglycoside kinase (APH)

APHs transfer the γ-phosphate of the nucleoside triphosphate cosubstrate to a hydroxyl nucleophile on the aminoglycoside substrate in the presence of magnesium ions (McKay et al., 1994). Collectively, the APH family can inactivate streptomycin, all disubstituted 2-deoxystreptamine aminoglycosides, as well as the atypical aminoglycosides spectinomycin and hygromycin (Table 1). It was thought that the canonical phosphate donor for these enzymes was ATP but it is now recognized that several aminoglycoside kinases use GTP instead of or in addition to ATP (Shakya and Wright, 2010; Frase et al., 2012; Shi and Berghuis, 2012).

**Table 1:** **Substrate profiles of APH enzymes**.

**APHs**	**Substrate**	**References**
APH(2″)-I (bifunctional enzyme)	Kanamycin, gentamicin, tobramycin, dibekacin, sisomicin, netilmicin, amikacin, isepamicin, neomycin, ribostamycin, paromomycin, lividomycin, butirosin	Ferretti et al., 1986; Daigle et al., 1999; Toth et al., 2009
APH(2″)-II	Kanamycin, gentamicin, tobramycin, dibekacin, sisomicin, netilimicin, amikacin, isepamicin, arbekacin	Kao et al., 2000; Toth et al., 2007, 2009
APH(2″)-III	Kanamycin, gentamicin, tobramycin, dibekacin, sisomicin, netilimicin	Chow et al., 1997; Badarau et al., 2008; Toth et al., 2009
APH(2″)-IV	Kanamycin, gentamicin, tobramycin, dibekacin, sisomicin, netilmicin, amikacin, isepamicin, arbekacin	Tsai et al., 1998; Toth et al., 2009
APH(3′)-I	Kanamycin, gentamicin, neomycin, ribostamycin, paromomycin, lividomycin	Matsuhashi et al., 1975; Oka et al., 1981; Pansegrau et al., 1987; Lee et al., 1991; Shaw et al., 1993
APH(3′)-II	Kanamycin, gentamicin, neomycin, ribostamycin, paromomycin, butirosin	Matsuhashi et al., 1975; Perlin and Lerner, 1981; Beck et al., 1982; Okazaki and Avison, 2007; Shaw et al., 1993
APH(3′)-III	Kanamycin, gentamicin, amikacin, isepamicin, neomycin, ribostamycin, paromomycin, lividomycin, butirosin	Lambert et al., 1985; Trieu-Cuot and Courvalin, 1986
APH(3′)-IV	Kanamycin, neomycin, ribostamycin, paromomycin, butirosin	Herbert et al., 1983, 1986
APH(3′)-V	Kanamycin, neomycin, ribostamycin, paramomycin	Thompson and Gray, 1983; Hoshiko et al., 1988; Salauze and Davies, 1991
APH(3′)-VI	Kanamycin, gentamicin, amikacin, isepamicin, neomycin, ribostamycin, praomomycin, butirosin	Lambert et al., 1988, 1990; Martin et al., 1988
APH(3′)-VII	Kanamycin, amikacin, neomycin	Tenover and Elvrum, 1988
APH(9)-I	Spectinomycin	Lyutzkanova et al., 1997; Suter et al., 1997
APH(4)-I	Hygromycin	Gritz and Davies, 1983; Rao et al., 1983
APH(7″)-I	Hygromycin	Pardo et al., 1985; Zalacain et al., 1986
APH(6)-I	Streptomycin	Distler et al., 1987; Shinkawa et al., 1987; Vögtli and Hütter, 1987
APH(3″)-I	Streptomycin	Heinzel et al., 1988; Ramon-Garcia et al., 2006

To date, over 40 crystal structures of 8 established or putative APH enzymes have been analyzed, covering APH(3′)-IIa (Nurizzo et al., 2003), APH(3′)-IIIa (Hon et al., 1997), APH(2″)-IIa (Young et al., 2009), APH(2″)-IIIa (Smith et al., 2012), APH(2″)-IVa (Shi et al., 2011), APH(4)-Ia (Stogios et al., 2011), APH(9)-Ia (Fong et al., 2010), and Rv3168 (Kim et al., 2011). Detailed structural information of the clinically important bifunctional enzyme AAC(6′)-Ie/APH(2″)-Ia remains elusive, but a low resolution SAXS model has given us some preliminary insight on the overall structure of this enzyme (Caldwell and Berghuis, 2012). In addition, crystal structures of APH(3′)-Ia and two putative aminoglycoside kinases have been released by various structure genomics centers and await further analysis (PDB accession numbers 2R78, 3CSV, 3DXP).

The collection of APH structures available demonstrates the diverse characteristics of this family of enzymes. APH(2″), with five members, and APH(3′) with seven members, are the two largest subfamilies of APHs (Ramirez and Tolmasky, 2010). Members of these subfamilies target different arrays of disubstituted 2-deoxystreptamine aminoglycosides and their prevalence among clinical isolates makes their structural information invaluable. APH(4) and APH(9) subfamilies each has only two members with a single substrate specificity. They are clinically less significant since neither enzyme is common in clinical isolates and hygromycin B, the substrate of APH(4) is used only in animals. Nonetheless, their crystal structures have provided important insights into the basis of substrate binding and specificity. The following sections summarize our structural understanding of the substrate binding sites of APH enzymes.

### Overall structure

**Figure 2:**
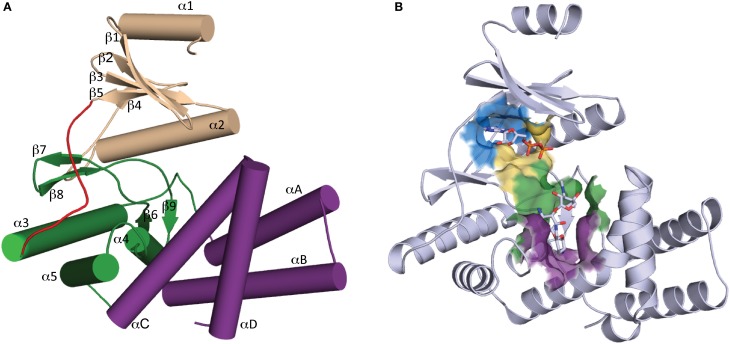
**Overall structure of APH. (A)** Secondary structure elements of a typical APH enzyme. The N-terminal lobe is colored tan, the hinge region is colored red, the core and helical subdomains in the C-terminal lobe are colored green and purple, respectively. **(B)** Cartoon representation of APH(2″)-IIa with ADP and gentamicin C1a in stick representation. The location of the active site is highlighted by a surface where the nucleoside pocket is colored blue, the triphosphate pocket is colored yellow, the catalytic pocket is in green, and the specificity pocket is in purple.

Determination of the first crystal structure of an aminoglycoside kinase enzyme, APH(3′)-IIIa, revealed a striking resemblance to the catalytic subunit of eukaryotic protein kinases (ePKs) (Hon et al., 1997). Elucidation of crystal structures of other members of the APH family confirm that APHs belong to the ePK superfamily of enzymes (Hanks and Hunter, 1995) (Figure 2), which also includes choline kinase and some lipid kinases (Scheeff and Bourne, 2005).

APH enzymes can be considered to have two lobes (Figure 2A): the N-terminal and the C-terminal, which are connected by a hinge segment. The N-terminal lobe consists of a 5-stranded antiparallel β-sheet flanked by a short helix in the N-terminus and a second longer α-helix located between β-strands 3 and 4. The C-terminal lobe is largely α-helical and can be further divided into the “core” and “helical” subdomains. The N-terminal lobe and the core subdomain are well conserved among APHs and ePKs and together they are involved in the binding of nucleoside triphosphate and catalysis. The helical subdomain is in contrast more variable in its composition and architecture and it provides the framework for aminoglycoside binding and recognition.

### Active site architecture

**Figure 3:**
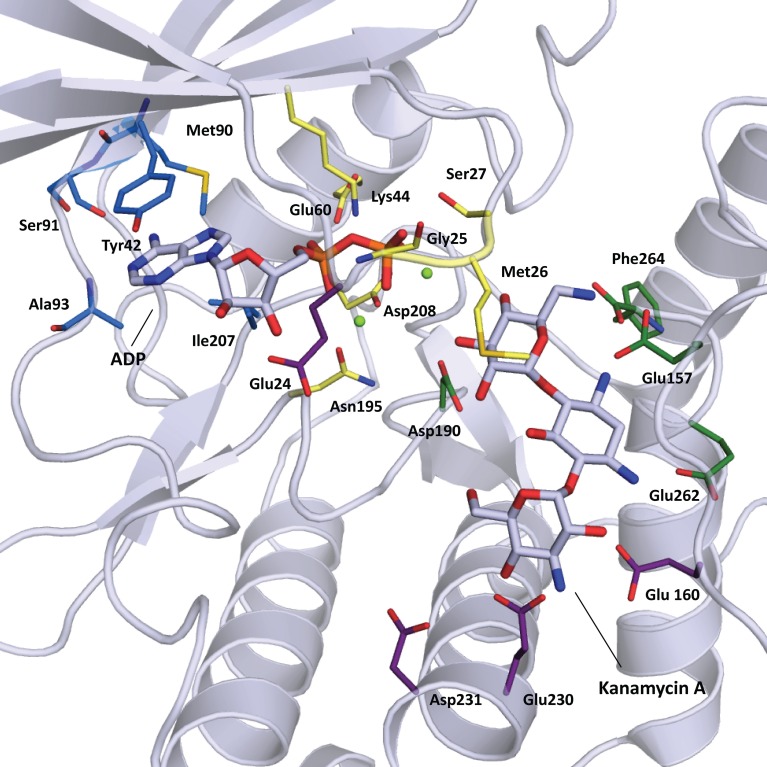
**Cartoon representation of APH(3′)-IIIa (1L8T) with ADP and kanamycin A in stick representation.** Residues important for substrate binding are displayed in stick representation and colored based on location: nucleoside pocket (blue), triphosphate pocket (yellow), catalytic pocket (green), and specificity pocket (purple). Two magnesium ions important for phosphate binding are shown as light green spheres.

The structural elements involved in nucleotide binding and aminoglycoside binding are largely conserved among all known APHs, despite significant amino acid sequence divergence. Based on the numerous substrate-bound structures now available, we can deconstruct the active site into four distinct subsites (Figure 2B): (1) the nucleoside pocket, (2) the triphosphate pocket, (3) the catalytic pocket, and (4) the specificity pocket. Structural elements paralleling those of ePKs are found in their respective pockets. Key residues in each subsite are summarized in Figure 3 and discussed in more details in the following sections.

#### Nucleoside pocket

**Figure 4:**
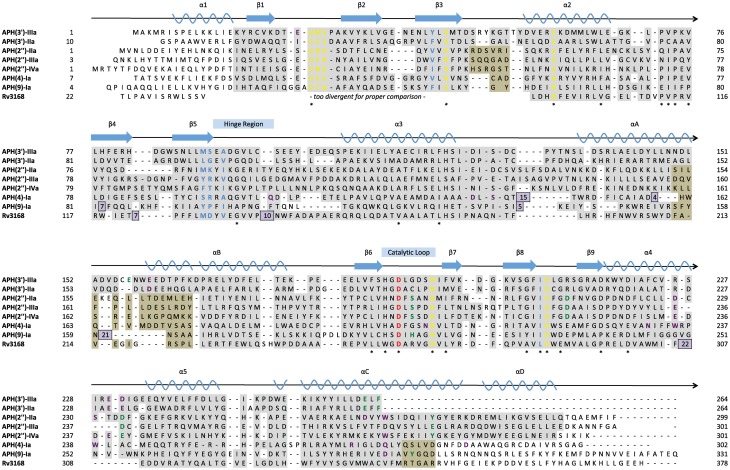
**Structure-based multiple sequence alignment of seven APH enzymes and Rv3168 (PDB codes: 1L8T, 1ND4, 3HAM, 3TDV, 3SG8, 3TYK, 3I0O, 3ATT).** Secondary structural elements are shown above the alignment with nomenclature corresponding to APH(2″) enzymes. Residues are color-coded based on location: nucleoside pocket (blue), triphosphate pocket (yellow), catalytic pocket (green), and specificity pocket (purple). The catalytic aspartate is highlighted in red. Conserved residues are indicated with a star. Residues with a gray background were found in structurally identical locations, and in cases where two subsets of enzymes have different structurally conserved elements, a tan background was used in addition to gray. Residues with a white background represent structural elements or conformations specific to a single enzyme. Inserts are shown as purple boxes, with the number displayed indicating the number of residues inserted at that location.

**Figure 5:**
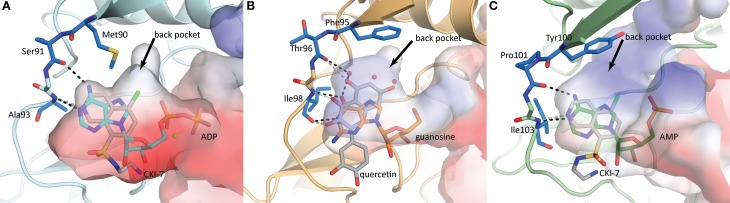
**Nucleoside binding pockets of (A) APH(3′)-IIIa (1L8T) with ADP (cyan), (B) APH(2″)-IVa (4DT9) with guanosine (orange), and (C) APH(9)-Ia (3I0Q) with AMP (green).** The binding modes of the nucleotide substrate and an ePK inhibitor [**(A,C)** CKI-7, **(B)** quercetin] are compared. For each panel, the gatekeeper residue and two hinge residues that form the ATP/GTP binding template are highlighted in blue stick representation. Hydrogen-bonding interactions between the hinge and the substrate are shown as black dotted lines and those between the hinge and the inhibitor are shown as gray dotted lines. The black arrows point to a hydrophobic back pocket in each binding site that is not normally occupied by the nucleotide substrate.

The nucleoside pocket is a cleft described by residues from strands 1, 2, and 3 of the β-sheet in the N-terminal lobe, two short β-strands and adjacent loop regions from the core subdomain, and the N-terminal segment of the hinge region. Consistent with the less polar character of the cofactor bound in this pocket, van der Waals interactions play an important role in stabilizing the nucleoside moiety, and the purine base is often sandwiched between hydrophobic sidechains projecting from opposite sides of the cleft [e.g., Tyr42 and Ile207 in APH(3′)-IIIa, Figure 3]. Few hydrogen-bonding interactions are observed, with those consistently found forming between the purine base and residues of the hinge region in a pattern reminiscent of that seen in Watson-Crick base pairing. Despite considerable sequence disparity (Figure 4), the structure of the nucleoside pocket is well conserved, partly because many interactions occur with the backbone atoms, which is particularly evident in the hinge region [Ser91 and Ala93 in APH(3′)-IIIa, Figure 3] Although the hinge region is an extended loop, it is thought to be relatively inflexible, and its exact conformation has been linked to controlling the nucleotide selectivity of APH enzymes (Shi and Berghuis, 2012). Indeed, it has been noted that the GTP-binding template is present even in ATP-specific APHs, but in these enzymes the hinge region assumes a conformation such that its orientation is not conducive for GTP-binding (Smith et al., 2012). Crystal structures of APHs with GTP analogs have shown that the guanine moiety tends to be bound less deeply in the cleft compared to adenine and stabilization by the hinge is crucial to its binding (Figure 5). In addition, the identity of a residue lining the interior of the cleft or the back pocket [Met90 in APH(3′)-IIIa, known as the gatekeeper residue in ePKs, Figure 3] plays a pivotal role in determining which nucleotide is preferred.

#### Triphosphate pocket

The triphosphate pocket connects the nucleoside pocket with the catalytic pocket and resembles an enclosed channel in most APH enzymes. Despite the negative charges present on the phosphate groups, there are very few basic residues lining this pocket, perhaps to mitigate any unfavorable electrostatic effects on the adjacent catalytic pocket. Instead, either one or a pair of magnesium ions are typically employed for the coordination of the phosphate moieties to conserved residues of the enzyme [Asn195 and Asp208 in APH(3′)-IIIa, Figure 3]. Upon nucleotide binding, a Lys residue interacts with the α- and β-phosphates and properly orients the triphosphate moiety for catalysis. This Lys residue [Lys44 in APH(3′)-IIIa, Figure 3] forms part of the highly conserved F/Y-x-K motif in both APHs and ePKs and interacts with a Glu residue from helix α 2 [Glu60 in APH(3′)-IIIa, Figure 3]. Interestingly, APH(2″)-IIIa, APH(4)-Ia and the putative aminoglycoside phosphotransferase Rv3168 from *Mycobacterium tuberculosis* do not have the F/Y-x-K motif and feature an Arg in the corresponding position instead. The nucleotide-bound crystal structures show that this residue is involved in an identical role in phosphate binding (Kim et al., 2011; Smith et al., 2012). An Arg in this position has occasionally been observed for ePKs. For most APH enzymes, a loop between strands β 1 and β 2, often referred to as the nucleotide-positioning loop, has been shown to undergo a large-scale rearrangement to fold over the bound cofactor (Burk et al., 2001). The nucleotide positioning loop is analogous to the glycine-rich G-loop found near the catalytic site of many ePKs and key residues in this loop, including Met26 and Ser27 in APH(3′)-IIIa (Figure 3), have been demonstrated to enhance phosphoryl transfer and contribute to a largely dissociative catalytic mechanism (Thompson et al., 2002).

#### Catalytic pocket

The catalytic subsite is situated at the interface between the core subdomain, the helical subdomain, and for some APHs also the N-terminal lobe. Residues forming this subsite have evolved to stabilize the aminoglycoside substrate such that the hydroxyl group intended for phosphorylation is positioned in close proximity to the catalytic loop containing an essential Asp residue [Asp190 in APH(3′)-IIIa, Figure 3] as part of the conserved H-x-D-x-x-x-x-N sequence, also known as the Brenner motif (Brenner, 1987). Since the ring bearing the target hydroxyl group differs among the APH subfamilies, the catalytic subsite is correspondingly diverse. In APH(3′) enzymes, this pocket binds the neamine core scaffold common to 4,5- and 4,6-disubstitued aminoglycosides (rings A and B) (Fong and Berghuis, 2002), whereas for the APH(2″) subfamily, this pocket binds the double prime ring or ring C in 4,6-disubstituted aminoglycosides (Young et al., 2009).

#### Specificity pocket

The specificity pocket is located deeper in the divide between the helical and core subdomains. In APH(4)-Ia, APH(2″) and APH(3′) enzymes this pocket is negatively charged, complementary to the cationic aminoglycoside substrates; whereas in APH(9)-Ia, this pocket is predominantly neutral corresponding to the non-polar nature of the spectinomycin substrate.

Although each subfamily of APH has a unique range of substrates, structural elements in this subsite are largely conserved. The binding of the aminoglycoside substrates are effected by interactions with residues located on the C-terminal portion of the catalytic loop, residues located on helix α 4 in the core subdomain, as well as those on helix α C in the helical subdomain. In APH(4)-Ia, APH(9)-Ia, and APH(2″) enzymes, the helical subdomain is composed of a relatively rigid four-helix bundle. It has been observed in APH(2″)-IVa and APH(9)-Ia that these enzymes undergo a conformation change upon the binding of the antibiotic that involves in part a small movement in the helical subdomain. It has been hypothesized that the limited flexibility of helices contribute to the formation of more rigid binding pockets and thus smaller substrate ranges for these subfamilies of APHs (Stogios et al., 2011). Indeed, for APH(2″) enzymes, favorable electrostatic interactions and a large number of hydrogen-bonds ensure that rings A and B are stabilized with very restricted conformational freedom (Shi et al., 2011). This limits the selectivity of the APH(2″) subfamily to 4,6-disubstituted aminoglycosides, because if 4,5-disubstituted aminoglycosides were bound in a catalytically competent manner, the neamine core scaffold would clash with the helical subdomain. Instead, the interactions of the specificity subsite are sufficiently strong that it is likely that when 4,5-disubstituted aminoglycosides bind to APH(2″) enzymes, the interactions with rings A and B would be maintained, thus causing ring C and the target hydroxyl group to be oriented away from the catalytic loop, which renders this class of aminoglycosides competitive inhibitors rather than substrates (Toth et al., 2010). Similarly, the specificity subsite in APH(4)-Ia or APH(9)-Ia could be considered so restrictive that only hygromycin B or spectinomycin, respectively, are recognized as substrates (Fong et al., 2010; Stogios et al., 2011). Notably, the C-terminal helix α D is significantly longer in APH(4)-Ia and APH(9)-Ia, and its position as well as the orientation of the helical subdomain as a whole give rise to a more compact and defined specificity pocket relative to APH(2″). In addition to the gross secondary structural elements, in APH(4)-Ia the single substrate specificity can also be attributed to the presence of hydrophobic residues in the specificity pocket that correspond to the unique twisted shape of hygromycin B; whereas in APH(9)-Ia, the polar groups of spectinomycin are complemented by localized polar areas in the neutrally charged specificity pocket.

In contrast, the specificity subsite of the APH(3′) subfamily contains distinct structural features that strongly influence the aminoglycoside selectivity of the enzyme. The APH(3′) enzymes are notable among the APHs due to their ability to phosphorylate both 4,5- and 4,6-disubstituted 2-deoxystretpamine aminoglycosides (Shaw et al., 1993). The specificity subsite of APH(3′) is comparatively spacious and can be seen as having two parts, one for binding 4,5-disubstituted aminoglycosides, and another for binding 4,6-disubstituted variants (Fong and Berghuis, 2002). Furthermore, APH(3′) enzymes have a reduced helical subdomain, containing only three helices (α A, α B, and α C). Most remarkably, however, is the loop insertion between α A and α B. The plasticity of the aminoglycoside binding site in APH(3′) enzymes can be partially ascribed to this flexible region termed the aminoglycoside-binding loop. This loop undergoes a conformation change upon the binding an aminoglycoside and folds toward the substrate, thereby completing the binding pocket and allowing a number of acidic residues in this loop to form hydrogen bonds with the substrate (Glu157 and Glu160, Figure 3). While the interactions between the substrates and residues on α C (Glu262 and Phe264, Figure 3) are critical (Thompson et al., 1999), the flexible aminoglycoside-binding loop makes it possible to customize the size and shape of the specificity pocket, allowing the enzyme to adapt to the structurally different substrates (Fong and Berghuis, 2009).

## Strategies to counteract enzyme-mediated resistance

Broadly, two strategies are considered in overcoming AME-mediated resistance. The first involves the development of novel antibiotic compounds that can evade inactivation by resistance enzymes; the second requires inhibition of the resistance mechanism such that the activity of existing aminoglycosides can be restored. Both of these approaches have precedents in the β-lactam class of antibiotics. The semi-synthetic β-lactam methicillin was the first antibiotic to successfully evade resistance by degradative enzymes (Sheehan, 1967). Methicillin differs from its parent compound in a side chain substitution on the 6-aminopenicillic acid core, resulting in a significant reduction in binding affinity to penicillinase enzymes and is therefore able to retain its antibiotic activity. Alternatively, penicillinase can be thwarted by β-lactamase inhibitors, which bind irreversibly to β-lactamase enzymes. Some have successfully been co-formulated with β-lactam antibiotics for the treatment of β-lactam-resistant infections (Drawz and Bonomo, 2010). For aminoglycosides, clinical success has resulted from development of aminoglycosides that evade resistance, similar to methicillin. However, aminoglycoside adjuvants acting as AME inhibitors have yet to progress to the clinic. The following sections summarize the strategies under investigation to counteract the activities of AMEs.

### Evading resistance

#### Removal of modifiable groups

Removal of functional groups of aminoglycosides targeted by AMEs has yielded some success in combating resistance. These modified compounds are able to elude inactivation by specific AMEs while retaining their ability to bind to the ribosome and exert their bactericidal effects. The semisynthetic dibekacin (3′,4′-dideoxykanamycin B) (Umezawa et al., 1971) was modeled after the naturally occurring tobramycin (3′-deoxykanamycin B) (Koch and Rhoades, 1970) to avoid AME-mediated modification. Both compounds lack the 3′-hydroxyl group, and are therefore useful against bacteria that harbor the *aph(3*′) resistance factor. Nonetheless, they can still be modified by AMEs with different regiospecificities. Furthermore, the feasibility of this strategy is limited since many of the functional groups of aminoglycosides are tightly linked to their antibacterial properties (Salian et al., 2012).

#### Steric hinderance

Butirosin, the naturally occurring 4,5-disubstituted aminoglycoside containing a (*S*)-4-amino-2-hydroxybutyrate (AHB) at position 1 of the 2-deoxystreptamine (Figure 1B), is an effective aminoglycoside that is resistant to the action of many AMEs. It is postulated that the bulky AHB interferes with binding to the active site of many AMEs (Fong and Berghuis, 2009). Consequently, an AHB group was added to kanamycin and dibekacin to produce amikacin (Kawaguchi et al., 1972) and arbekacin (Kondo et al., 1973), respectively (Figure 1A). While the introduction of 3-amino-2-hydroxypropionate (AHP) to gentamicin B produces isepamicin (Nagabhushan et al., 1978), the addition of an ethyl group to sisomicin generates netilmicin (Wright, 1976) (Figure 1A). These derivatives have been shown to be clinically useful. Notably, arbekacin has been a success in Japan for the treatment of MRSA infections (Kondo and Hotta, 1999) as it lacks the modifiable hydroxyl groups at positions 3′ and 4′ and its antibiotic activity is unaffected by acetylation at the 2′- or 3″-positions (Hotta et al., 1996, 1998). In addition to providing protection against AMEs, the AHB group also contributes to the binding to its target site in the ribosome. The crystal structure of amikacin bound to the decoding center revealed that amikacin binds the A-site in the same fashion as its parent compound kanamycin, supplemented with favorable contacts formed by the AHB (Kondo et al., 2006).

Analogous to the N1-acylated aminoglycosides, Shaul et al. recently reported on the generation of 6′- and 6‴-acylated tobramycin and paromomycin (Shaul et al., 2011). Some derivatives were able to exert antibiotic activity in the presence of some AMEs. However, unlike the derivation at position N1 of the 2-deoxystreptamine that has been successfully applied to a range of aminoglycosides, only specific permutations of selective aminoglycosides and acyl groups substituted at the 6′- or 6‴-position give rise to aminoglycoside derivatives that are active against bacterial strains carrying various AMEs.

Capitalizing on the crystal structures of the ribosome in complex with various aminoglycosides as well as the success of aminoglycoside derivatives with an acyl substitution at position 1 of the 2-deoxystreptmaine ring and lacking selected modifiable groups, two analogous novel aminoglycosides have been developed. Plazomicin (previously ACHN-490), a “neoglycoside,” represents the leading candidate for “next-generation” aminoglycosides. It is derived from sisomicin, with the addition of an AHB group at position 1 of the 2-deoxystreptamine and a hydroxyethyl group at position 6′. These two modifications, combined with the inherent lack of 3′- and 4′- hydroxyl groups in sisomicin combine to produce an aminoglycoside that is resistant to all tested aminoglycoside resistance enzymes with the exception of AAC(2′) enzymes, a resistance factor that has not been observed in clinical strains (Armstrong and Miller, 2010). In early 2012, Phase II clinical trials have successfully been completed for the treatment of complicated urinary tract infections and acute pyelonephritis, without the nephrotoxicity or ototoxicity often associated with aminoglycoside therapy (Cass et al., 2011). A related molecule 2″-*O*-(phenethylamino)ethyl- and 1-*N*-AHB-substituted 3′,4′-dideoxy paromomycin showed potent antibiotic activity against a range of resistant bacteria including vancomcyin resistant *S. aureus* (VRSA) and vancomycin intermediate *S. aureus* (VISA) strains (Hanessian et al., 2010). Moreover, the crystal structure of this variant paromomycin with an RNA fragment containing the A-site revealed that it has an altered binding mode compared to the parent paromomycin compound (Kondo et al., 2007).

A new generation of aminoglycosides would promise a feasible path in the mitigation of the effects of AMEs in resistant bacteria. However, coevolution of AMEs will likely accompany the introduction of novel aminoglycoside variants into the therapeutic regime thus jeopardizing the longevity and effectiveness of these new drugs. Such a phenomenon can be exemplified by a variant of aminoglycoside acetyltransferase AAC(6′)-Ib in which a change in two base pairs in its gene was sufficient to extend the enzyme's substrate specificity to include ciprofloxacin, a synthetic antibiotic belonging to the fluoroquinolone class, resulting in a decreased susceptibility to ciprofloxacin (Robicsek et al., 2006).

### Current approaches to APH inhibitor discovery

Thus far, attempts at developing a potent and specific inhibitor against APHs have not been successful. Given our growing understanding of the structure and mechanism of APHs, a strong case can be made for structure-guided design of an inhibitor, an approach that has not been explored for finding inhibitors for this class of enzymes.

An ideal APH inhibitor would universally target most if not all members of the APH family with high affinity and specificity such that little to no cross-reactivity with ePKs or other human enzymes would occur. Although the prospect of such a pan-APH inhibitor is poor, it is not unrealistic to aim for the development of an inhibitor that can effectively target a selection of APHs such as members of the same subfamily. In general, Figure 4 shows that residues forming the triphosphate pocket are the most conserved among the APHs. However, accessibility of this subsite is poor and the sequence conservation also extends to ePKs, thus suggesting that while targeting this subsite may yield an inhibitor capable of acting against multiple APHs, selectivity toward resistance enzymes will be difficult to obtain. On the other hand, the large variations in the aminoglycoside-binding site, reflective of the diverse substrate-profiles and regiospecificity of APHs (Table 1), leaves little opportunity for a common inhibitor, thereby leaving the nucleoside pocket the most promising site of inhibition. This subpocket has served as a starting point for many structure-guided inhibitor design studies since this pocket at once shows strong conservation among APHs and bears small but significant differences with ePKs, which offer opportunities for specificity.

#### Adapting ePK inhibitors

**Figure 6:**
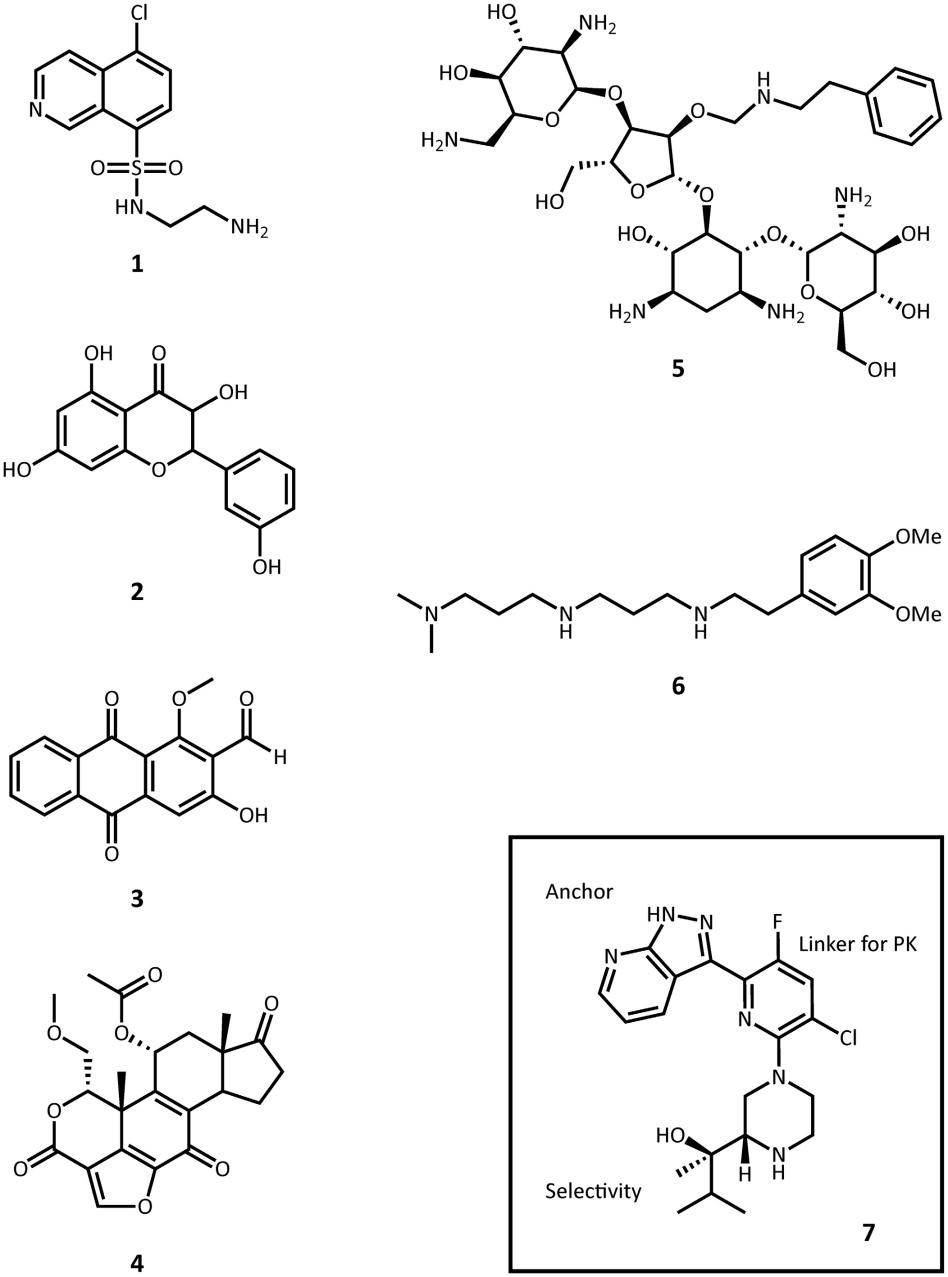
**Representative structures small molecule inhibitors. 1**, CKI-7; **2**, quercetin; **3**, damnacanthal; **4**, wortmannin; **5**, paromomycin O2″-ether analog; **6**, non-carbohydrate diamine inhibitor; **7**, protein kinase C θ inhibitor.

ePKs are of significant medical relevance due to their central roles in signal transduction and regulatory pathways. Deregulation or overexpression of ePKs plays a role to many diseases, including diabetes and some cancers. As a consequence, a plethora of ePK inhibitors have been developed, most of which target the ATP-binding pocket. Soon after the structural homology between APHs and ePKs was uncovered, several known inhibitors of ePKs were assayed for their activity toward APHs. Isoquinolinesulfonamide derivatives, notably CKI-7 (**1**, Figure 6), were among the first compounds discovered to inhibit some APHs such as APH(3′)-IIIa (Daigle et al., 1997). Recently, a more comprehensive screening for APH inhibitors was carried out by Shakya *et al*., who tested a set of 14 aminoglycoside and macrolide phosphotransferases against a library of 80 protein kinase inhibitors with diverse chemical scaffolds (Shakya et al., 2011). The resulting data allowed the authors to construct a small molecule discrimination map of the most commonly encountered APH enzymes. It was discovered, for instance, that some ePK inhibitors such as quercetin (**2**) or damnacanthal (**3**) were active against multiple APHs, while some APHs, including the clinically relevant APH(2″)-Ia, was virtually unaffected by all compounds tested. Interestingly, APH(2″)-Ia was shown to be inhibited by CKI-7 in the previous study (Daigle et al., 1997). A natural next step would be to gain better understanding of the specific mode of binding of the more promising leads identified thus far and explore distinctive structural features in order to modify the inhibitors for improved specificity. Recently, the crystal structures of APH(3′)-IIIa and APH(9)-Ia in complex with CKI-7 were determined, representing the first co-crystal structures of an APH with an active small molecule inhibitor (Fong et al., 2011). More ePK inhibitor-bound structures of APH have since been solved, including one of APH(2″)-IVa bound with the wide-spectrum flavonol type inhibitor, quercetin (**2**) (Shakya et al., 2011), as well as a series of ePK inhibitor-bound structures APH(3′)-Ia solved by the Center for Structural Genomics of Infectious Diseases (PDB accession numbers 4GKH, 4GKI, 4FEU, 4FEV, 4FEW, 4FEX).

Crystal structures of APH-CKI-7 complexes solved by Fong et al. show that the inhibitor-bound enzyme conformation largely parallels that of the ATP-bound state (Fong et al., 2011). A notable difference is observed in the conformation of the region termed the glycine-rich loop in ePKs [res. 21-27 in APH(3′)-IIIa], which in the inhibitor-bound complex resembles more the apo APH structures than the nucleotide-bound structures. The equivalent region in APH(2″)-IVa adopts the same conformation in the apo, adenosine-bound, and the quercetin-bound structures (Shakya et al., 2011; Shi et al., 2011; Shi and Berghuis, 2012). The binding mode of the inhibitors are comparable to that of the adenine ring, making van der Waals interactions with the non-polar side chains and hydrogen bond interactions with the main chain atoms in the hinge regions. Further analyses on the structure-activity relationship between various ePK inhibitors and APHs confirm the critical nature of these hydrogen bond interactions (Sowadski et al., 1999; Fong et al., 2011; Shakya et al., 2011). Comparative analysis with the crystal structure of CKI-7 bound to the ePK casein kinase 1 revealed many similarities in the mode of inhibitor binding. However, a number of subtle distinguishing features in the nucleotide-binding site of APH enzymes, such as the conformation of the hinge region and the presence of a large aromatic residue capable of forming stacking interactions with the isoquinoline portion of the inhibitor, can serve as starting points for the development of APH-specific inhibitors. Though many types of ATP-competitive inhibitors developed for ePKs show activity against APHs, their inhibition constants are invariably significantly higher, which by itself suggests that binding pockets of APHs are still sufficiently different from most ePKs to allow for more specific targeting. This is corroborated by the crystal structures of quercetin-bound APH(2″)-IVa (Shakya et al., 2011) and various ePKs (PDB accession numbers 1E8W, 2HCK, 2O3P, and 3LM5) in which the inhibitor binds to each kinase in a unique manner as a result of the different interactions effected by the non-conserved elements in the nucleoside-binding pocket.

In a similar vein, some lipid kinases are known to share a comparable fold with ePKs and APHs, and the PI-3 kinase inhibitor wortmannin (**4**) has been shown to inhibit APH(2″)-Ia (Boehr et al., 2001). A prominent concern of this translational approach is of course the specificity of the inhibitor. As the APH-CKI-7 crystal structures demonstrate, the inhibitor's mode of binding closely resembles that seen for ePKs such as casein kinase 1. More structural information will prove instrumental in the development of specific APH inhibitors adapted from ePK or lipid kinase inhibitor scaffolds that minimize cross-reactivity and undesired side effects.

#### Modifying existing aminoglycosides

Many studies have been published on the modification of existing aminoglycosides to generate APH inhibitors that blocks the aminoglycoside-binding pocket. These efforts, which include aminoglycoside dimers, and aminoglycoside-small molecule conjugates, have been excellently reviewed elsewhere and will not be recapitulated here (Houghton et al., 2010). A recent effort in this direction deserves mentioning, however: Szychowski et al. have developed a series of paromomycin analogs featuring hydrophobic substituents at the O2″ position (**5**, Figure 6) (Szychowski et al., 2011). These compounds have been shown to be active against aminoglycoside sensitive strains but not their resistant counterparts. Notably, they are not phosphorylated by APH(3′)-IIIa and they act competitively with respect to the aminoglycoside substrate and mixed uncompetitively with respect to ATP in the inhibition of APH(3′)-IIIa and AAC(6′)-Ii. Unfortunately, they do not demonstrate any synergistic effects with amikacin or paromomycin against aminoglycoside-resistant strains.

In another study, Welch et al. used the 2-deoxystreptamine core as a starting point to synthesize a series of non-carbohydrate aminoglycoside analogs that extends to the phosphate-binding site and thus show competitive inhibition toward ATP-binding in several AMEs, including APH(3′)-IIIa and ANT(2″)-Ia (**6**) (Welch et al., 2005).

#### Uncompetitive inhibition

At this time, alternative inhibition mechanisms with small molecules other than competitive inhibitors have not been extensively explored in APH enzymes. However, known inhibitors of kinases are by no means limited to small molecules that target the ATP-binding site. Recent pre-steady state kinetic studies on APH(3′)-IIIa have confirmed that ADP release is the rate-limiting step in the reaction mechanism (Lallemand et al., 2012). Theoretically, compounds that stabilize the enzyme-ADP complex could serve as uncompetitive inhibitors and promise an alternative route in the search of an effective APH inhibitor. In another study, Boehr et al. probed cationic peptides for their ability to inhibit aminoglycoside resistance enzymes on the basis that functional studies have shown that APHs can phosphorylate positively charged peptides. Several peptide candidates were identified, including indolicidin, with potencies in the micromolar range (Boehr et al., 2003). Steady-state kinetic analyses determined with APH(3′)-IIIa and APH(2″)-Ia demonstrated non-competitive inhibition patterns with respect to both the nucleotide and the aminoglycoside substrate, which suggest that the cationic peptides bind to APHs in a mode more complex than simply competing with the aminoglycoside for its negatively charged binding site. Although cationic peptides with antimicrobial properties remain an active field of investigation (Hancock and Sahl, 2006; Pompilio et al., 2012), no further study has been reported on their inhibitory effect on resistance enzymes. Structural information detailing their mode of binding would greatly advance our understanding of their mechanism of action and preserve cationic peptides as a potential avenue for adjuvant development.

## Outlook for APH inhibitor development

Although adapting ePK inhibitors presents a promising avenue for the discovery of inhibitors of APHs, most if not all ePK inhibitors were designed based on the scaffold for ATP-binding, possibly limiting their adaptability to those APH enzymes that utilize GTP as its phosphate source. Nonetheless, building on the extensive efforts invested in the development of protein kinase inhibitors over the past several decades, a structure-guided approach of designing APH inhibitors could begin with the ATP/GTP-binding templates of the hinge region. The general purine-binding template consists of three groups capable of forming hydrogen bonds with the ligand: the carbonyl group of a residue in the N-terminus of the hinge [Ser91 in APH(3′)-IIIa], and the amide nitrogen as well as the carbonyl group of the residue two amino acids C-terminal to the first [Ala93 in APH(3′)-IIIa] (Figure 5). Since all three groups form part of the backbone, this template is conserved for all APHs despite poor sequence similarity of the hinge region. However, it should be noted that the relative orientation of the groups can vary for different APH enzymes depending on their nucleotide specificity, since only a subset of two functional groups are involved in interacting with either the adenine or guanine moiety. To complement the purine-binding template, the inhibitor should contain a relatively inflexible “head group” with at least one hydrogen-bond donor and one acceptor that serves to anchor the molecule in the purine-binding pocket. The inhibitor could then be extended into the nucleoside pocket in two directions. The most apparent space is that normally occupied by the ribose moiety. Many ePK inhibitors, including CKI-7, take advantage of this pocket and interact with residues on the catalytic loop C-terminal to the catalytic aspartate [Ser194 in APH(3′)-IIIa]. Intriguingly, space-filling models of multiple APHs with a bound ATP-analog including APH(3′)-IIIa, APH(3′)-IIa, APH(2″)-IVa, and APH(9)-Ia show a hydrophobic pocket toward the interior of the binding cleft near atoms N7 and C8 of the adenine base (denoted by a black arrow in Figure 5) that might be exploitable for increased specificity and potency. In ePKs, this pocket is termed the back pocket and its accessibility greatly depends on the bulk of the gatekeeper residue (Zuccotto et al., 2010). Interestingly, the influence of the gatekeeper residue seems to be of reduced importance in APH enzymes, where APH(2″)-IIa (with Met85 as gatekeeper residue) has virtually no accessible back pocket while APH(9)-Ia (with Tyr100 as gatekeeper residue) has an extensive back pocket due to the more open orientation of its N-terminal β-sheet with respect to the binding cleft. The possibility of targeting this hydrophobic cavity, coupled with the relatively low sequence conservation and a high level of structural plasticity in this region, offers valuable opportunities to obtain selectivity. Finally, variations can be introduced to the intermediate portion of the molecule linking the head group with the selectivity fragment to optimize the pharmacokinetic properties of the small molecule.

A similar approach to inhibitor development involving a head group as anchor, an end group for selectivity, and a linker for pharmacokinetics has been successfully applied to optimizing a number of protein kinase inhibitors. Most recently, such efforts have led to the discovery of a protein kinase C θ inhibitor with strong *in vivo* potency in mice (**7**, Figure 6) (Jimenez et al., 2013).

Despite the above, the growing body of structural knowledge of different subfamilies of APH enzymes indicates that the development of a universal APH inhibitor not recognized by ePKs may not be achievable. In fact, the level of differences among the APH subfamilies is on par with or greater than those between APHs and their nearest ePK homologs. The diversity in the active site architecture of APH proteins, evidenced by the distinctive nucleotide and aminoglycoside substrate profiles for each individual member, suggests that pursuing selective inhibitors for the different subfamilies rather than a broad-spectrum inhibitor may be more fruitful. Realistically, researching specific inhibitors that target less widespread, atypical aminoglycoside resistance enzymes which inactivate single drugs, such as APH(9)-Ia or APH(4)-Ia, will unlikely prove profitable and complications due to these enzymes may be more efficiently overcome by developing modified aminoglycosides. However, in the absence of a healthy pipeline for next-generation aminoglycosides that circumvent resistance enzymes and given the ever-growing spread of resistance to existing aminoglycosides due to several key broad-spectrum enzymes, notably APH(3′)-IIIa and AAC(6′)/APH(2″), developing specific inhibitors that target the ATP- and GTP-binding capabilities of these enzymes still present invaluable steps of progress in the struggle against aminoglycoside resistance.

### Conflict of interest statement

The authors declare that the research was conducted in the absence of any commercial or financial relationships that could be construed as a potential conflict of interest.
